# The effects of precisely meeting estimated daily energy and lysine requirements for gestating sows over three consecutive pregnancies on sow reproductive and lactation performance

**DOI:** 10.1093/tas/txab226

**Published:** 2021-12-09

**Authors:** Victoria Stewart, R Quincy Buis, Brenda Christensen, Lauren L Hansen, Cornelis F M de Lange, Ira B Mandell, Lee-Anne Huber

**Affiliations:** Department of Animal Biosciences, University of Guelph, Guelph, Ontario, N1G 2W1, Canada

**Keywords:** electronic sow feeders, energy, gestating sows, lysine, precision feeding

## Abstract

The objective of the current study was to determine the effects of precisely meeting estimated daily energy and Lys requirements for gestating sows over three consecutive pregnancies on sow reproductive and lactation performance. A total of 105 sows (initial reproductive cycle 1.4 ± 0.5) were randomly assigned to a precision (PF; *n* = 50) or control (CON; *n* = 55) feeding program between days 2 and 9 of gestation and housed in group-pens equipped with electronic sow feeders capable of blending two diets. The PF sows received unique daily blends of two isocaloric diets (2518 kcal/kg NE; 0.80% and 0.20% standardized ileal digestible [SID] Lys, respectively), whereas CON sows received a static blend throughout gestation to achieve 0.56% SID Lys. After weaning, sows were re-bred and entered the same feeding program as in the previous pregnancy for two subsequent pregnancy cycles (PF: *n* = 36; CON: *n* = 37; average reproductive cycle: 2.4 ± 0.5; PF: *n* = 25; CON: *n* = 24; average reproductive cycle: 3.5 ± 0.5). Sows on the PF program received 97%, 105%, and 118% (average over three pregnancy cycles) of dietary energy and 67%, 79%, and 106% of SID Lys intakes compared to CON between days 5 and 37, 38 and 72, and 73 and 108 of gestation, respectively. Estimated N (26.1%) retention did not differ between gestation feeding programs in any pregnancy, but excess N excretion was less (1617 vs. 1750 ± 54 g/sow; *P* < 0.01) for PF vs. CON sows. Regardless of pregnancy cycle, sows that received the PF program had greater ADG between days 38 and 72 (614 vs. 518 ± 63 g/d; *P* < 0.05) and between days 73 and 108 (719 vs. 618 ± 94 g/d; *P* = 0.063) of gestation, and greater loin depth gain between days 63 and 110 of gestation (0.7 vs. −1.1 ± 1.6 mm; *P* < 0.05), but BW (235.1 kg) and backfat (17.8 mm) and loin (70.5 mm) depths on day 110 of gestation did not differ. The number of piglets born alive, stillborn, and mummified, and litter birth weight (16.5 kg) did not differ in any pregnancy cycle, nor did piglet ADG during lactation (250 g/d) and piglet BW (6.7 kg) at weaning. Sows that received the PF program during gestation had lower ADFI during lactation (5.7 vs. 6.2 ± 0.2 kg; *P* < 0.01). Therefore, using feeding programs that precisely match estimated daily energy and Lys requirements for gestating sows provides the opportunity to reduce N losses to the environment and reduce lactation feed usage, without negatively affecting sow reproductive and lactation performance.

## INTRODUCTION

The nutrient and energy requirements for gestating sows are not constant throughout the gestation period or across parities. For example, the estimated Lys requirement for a sow in the first reproductive cycle increases by 200% between days 0 and 114 of gestation, whereas the estimated energy requirement increases by 45% within the same timeframe (NRC, 2012). Furthermore, sows in the first reproductive cycle require nutrients and energy for maternal growth, whereas multi-parity sows have greater energy requirements for maintenance and must recuperate protein and energy stores that were mobilized in the previous lactation (NRC, 2012). In both of the aforementioned cases, sows must also support protein deposition in fetal, placenta, mammary gland, and uterine pools, which occur at different points in the gestation period and at different rates; the demand for protein deposition is influenced by litter size and is the major driver for (daily) amino acid requirements (Dourmad et al., 2008; NRC, 2012). Therefore, providing a constant quantity of a gestation diet with static nutrient composition is not sufficient to account for the unique daily nutrient and energy requirements for individual sows throughout gestation and across parities (e.g., Samuel et al., 2012; Thomas et al., 2021).

The sow is adept at buffering moderate nutrient and energy deficiencies and excesses in the diet (e.g., Bee, 2004; Lawlor et al., 2007; reviewed by Campos et al., 2012). However, under- and over-supplying energy can result in suboptimal body condition, which have negative implications for milk production during the lactation period, subsequent reproductive performance, and sow longevity (Dourmad et al., 1994; Young et al., 2004; Cerisuelo et al., 2010). In addition, under-supplying amino acids, particularly in late gestation when amino acid requirements are high, can lead to maternal body protein mobilization even before the onset of lactation, and also negatively affect subsequent reproductive performance (Lawlor et al., 2007; Thomas et al., 2021). Finally, over-supplying energy and nutrients to sows during gestation increase feed costs, as well as nutrient losses into the environment. Although phase- or parity-segregated feeding programs provide some flexibility for more closely matching estimated nutrient and energy requirements for groups of sows, there is further opportunity to improve precision by using electronic sow feeders (ESF) with feed blending capabilities to meet estimated daily nutrient and energy requirements for individual sows (Buis, 2016).

Therefore, the aim of the current study was to determine the long-term effects of precisely meeting estimated daily Lys and energy requirements for individual sows on each day of gestation across three consecutive pregnancies on reproductive and lactation performance.

## MATERIALS AND METHODS

### Animals and Housing

The experimental protocol was approved by the University of Guelph Animal Care Committee and followed Canadian Council on Animal Care guidelines (CCAC, 2009; AUP #3237). The study was conducted at the Arkell Swine Research Station (OMAFRA, University of Guelph, Arkell, ON, Canada).

One hundred five sows (62 Yorkshire and 43 Yorkshire × Landrace; average initial reproductive cycle 1.4 ± 0.5) were placed into one of four pens equipped with ESF 5 ± 3 d after breeding over five breeding batches (blocks). Sows were randomly assigned to one of two gestation feeding programs in the first gestation cycle: precision feeding (PF) or standard feeding (CON; *n* = 50 and 55, respectively). Sow genetics were distributed equally between treatments and sows were randomly bred with either Yorkshire, Landrace, or Duroc semen. The ESF [modified according to Buis (2016); Canarm, Arthur, ON, Canada] was supplied by two feed lines and had the ability to dispense precise amounts for each of two diets. Prior to entry, sows were weighed and tagged in one ear with a reusable radio frequency identification responder (RFID; half-duplex signaling technology; Allflex technologies, St-Hyacinthe, QC, Canada). Sows were allowed to navigate the ESF independently for 3 d. Research personnel manually guided sows that did not consume a meal during the 3-d adaptation period (~ 50% of sows) through the system on alternating days until sows began entering the ESF independently; most sows were assisted a maximum of three times. One sow did not learn to use the system and was removed from the study after 2 wk. Sows were confirmed pregnant 4 wk after insemination via ultrasound and those not pregnant were removed from the study. On day 110 ± 1 of gestation, sows were moved to farrowing crates. The number of piglets born alive, stillborn, and mummified was recorded and litters were standardized to between 10 and 12 piglets per litter based on piglet availability and only fostering piglets among sows within the same gestation feeding program. Piglets were processed according to farm standard operating procedure (i.e., weighing, tail docking, ear notching, needle teeth clipping, and iron dextran injection) within 24 h of birth. Males were surgically castrated at 4 d of age. Beginning on day 7 after birth, piglets were given ad libitum access to a commercial creep feed (Floradale Feedmill Ltd, Floradale, Ontario, Canada) and weaning occurred after a 20 ± 3 d lactation period. Sows were re-bred 5 ± 1 d after weaning and entered the same feeding program as in the previous pregnancy. Sows remained enrolled in the study for three consecutive gestation cycles. Throughout the study, sows were removed due to lameness, reproductive failure, illness, and death. Seventy-three sows completed the second pregnancy (i.e., were bred, confirmed pregnant, and farrowed; 36 PF and 37 CON sows including 41 Yorkshire and 32 Yorkshire × Landrace; 2.4 ± 0.5 average reproductive cycle) and forty-nine sows completed the third pregnancy (25 PF and 24 CON sows with 29 Yorkshire and 20 Yorkshire × Landrace; 3.5±0.5 average reproductive cycle). The experiment was conducted over a 20-mo period.

### Dietary Treatments and Feeding

The ESF has the capability to blend two basal diets and were each calibrated weekly by dispensing two 500-g allotments of each diet in each ESF to ensure that the screw auger rotation cycles dispensed correct amounts of each diet (Buis, 2016). The two basal diets were isocaloric (2518 kcal/kg NE) but were formulated to contain high or low protein contents (0.80% and 0.20% standardized ileal digestible [SID] Lys, respectively; Table 1). The CON sows received a constant blend of 1.32 kg of the high protein (HP) diet and 0.88 kg of the low protein (LP) diet on each day of gestation to mimic a conventional industry feeding program. For the precision feeding program, the NRC (2012) Nutrient Requirements Gestating Sow Model was used to estimate nutrient requirements on each day of gestation for each individual sow using body weight and reproductive cycle (parity; at the time of entry into group housing for each pregnancy) and estimated litter size (13.5) and piglet birth weight (1.4 kg). The model then determined the amount of the basal diets to blend together on each day of gestation to best match daily estimated energy and Lys requirements. The model was modified to keep daily maternal lipid deposition constant within each reproductive cycle (105, 96, 80, and 28 g/d in reproductive cycles 1, 2, 3, and 4, respectively) to ensure energy intake was above estimated sow requirements for maintenance and reproduction (Buis, 2016). Both the CON and PF blends met or exceeded the amino acid-to-Lys ratios recommended by the NRC (2012), both throughout gestation and across pregnancy cycles. The recipe was then uploaded to the ESF system (via PigCHAMP, Ames, IA), which was linked to the individual sow RFID so that when the sow entered the ESF system, the appropriate feed blend was dispensed on each day. The average daily feed blends between days 5 and 37, 38 and 72, and 73 and 108 for each pregnancy cycle are presented in Table 2. Sows on the PF program received 97%, 105%, and 118% (average over three pregnancy cycles) of dietary energy intakes and 67%, 79%, and 106% of SID Lys intakes compared to CON sows between days 5 and 37, 38 and 72, and 73 and 108 of gestation, respectively (Table 2). The ESF dispensed the sow’s daily feed allowance in multiple portions set 30 s apart based on the weight of the total feed allowance. Thus, regardless of feeding program, sows were able to consume the daily feed allotment in one meal or over multiple visits to the feeder during each daily feeding cycle. There was no carryover of missed feed allotments among daily feeding cycles. Feed usage for each sow during gestation was generated from the feed recipes.

**Table 1.** Ingredient composition and nutrient contents for high and low protein gestation diets and standard lactation diet (as-fed basis)^1^

Item	High protein	Low protein	Standard lactation
Ingredient composition, %			
Corn	69.01	86.37	48.41
Soybean meal	25.71	0.60	21.60
Barley	–	–	10.00
Wheat	–	–	12.50
Soybean hulls	–	7.50	–
Animal and vegetable fat blend	1.90	1.71	3.20
Limestone	1.46	1.32	1.65
Mono-calcium phosphate	1.15	1.70	0.75
Sodium chloride	0.27	0.30	0.49
Vitamin and mineral mix^2^	0.50	0.50	–
Commercial micro premix^3^	–	–	1.40
Total	100.00	100.00	100.00
Calculated nutrient contents			
NE, kcal/kg	2519	2519	2520
Crude protein, %	17.97	8.19	16.74
Total Lys, %	0.95	0.30	0.95
SID Lys, %^4^	0.80	0.20	0.74
Total calcium, %	0.92	0.92	0.86
Total phosphorus, %	0.65	0.65	0.55
Analyzed nutrient contents, %^5^			
Crude protein	18.70	9.33	16.93
Calcium	0.87	0.92	0.86
Phosphorus	0.65	0.66	0.55

^1^ Sows were fed a blend of high and low protein diets between gestation days 5 ± 2.5 and 110 ± 1.4 and a standard lactation diet between gestation day 110 ± 1.4 and weaning.

^2^ Provided per kg of premix: vitamin A, 2,000,000 IU as retinyl acetate; vitamin D_3_, 200,000 IU as cholecalciferol; vitamin E, 8,000 IU as dl-α-tocopherol acetate; vitamin K, 500 mg as menadione; pantothenic acid, 3,000 mg; riboflavin, 1,000 mg; choline, 100,000 mg; folic acid, 400 mg; niacin, 5,000 mg; thiamine, 300 mg; pyridoxine, 300 mg; vitamin B_12_, 5 mg; biotin, 40 mg; Cu, 3,000 mg from CuSO_4_×5H_2_O; Fe, 20,000 mg from FeSO_4_; Mn, 4,000 mg from MnSO_4_; Zn, 21,000 mg from ZnSO_4_; Se, 60 mg from Na_2_SeO_2_ and I, 100 mg from KI (DSM Nutritional Products Canada Inc., Ayr, ON, Canada).

^3^ Provided per kg of diet: vitamin A, 11000 IU; vitamin D, 1500 IU; vitamin E, 65 IU; Se, 0.3 mg; Cu, 25 mg; Zn, 150 mg; Fe, 175 mg; Mn, 27.5 mg; and I, 0.6 mg (Floradale Feed Mill Limited, Floradale, ON, Canada).

^4^ Standardized ileal digestible.

^5^ Values reflect the mean analysis of composite samples collected during six 3.5-mo intervals.

**Table 2.** Gestation feed blends and apparent energy and lysine intakes for sows that received a precision (PF) or static (CON) feeding program during gestation over three consecutive pregnancies

	Pregnancy 1		Pregnancy 2		Pregnancy 3		PF, % of CON^1^		
Item	PF^2^	CON^2^	PF	CON	PF	CON	Preg^3^ 1	Preg 2	Preg 3
No.^4^	50	55	36	37	25	24			
High protein feed allowance, g/d									
Days 5 to 37	887	1320	645	1320	423	1320	67.2	48.9	32.0
Days 38 to 72	1133	1320	850	1320	578	1320	85.8	64.4	43.8
Days 73 to 108	1695	1320	1274	1320	946	1320	128.4	96.5	71.7
Low protein feed allowance, g/d									
Days 5 to 37	1144	880	1574	880	1758	880	130.0	178.9	199.8
Days 38 to 72	1080	880	1528	880	1732	880	122.7	173.6	196.8
Days 73 to 108	851	880	1399	880	1622	880	96.7	159.0	184.3
Net energy intake, kcal/d									
Days 5 to 37	5116	5542	5589	5542	5497	5542	92.3	100.8	99.2
Days 38 to 72	5576	5542	5989	5542	5826	5542	100.6	108.1	105.1
Days 73 to 108	6413	5542	6732	5542	6474	5542	116.9	121.5	116.8
SID lysine intake, g/d^5^									
Days 5 to 37	9.4	12.3	8.3	12.3	6.9	12.3	76.4	67.5	56.1
Days 38 to 72	11.2	12.3	9.9	12.3	8.1	12.3	91.1	80.5	65.9
Days 73 to 108	15.3	12.3	13.0	12.3	10.8	12.3	124.4	105.7	87.8

^1^PF sow values as a percentage of CON sow values.

^2^ PF sows received unique daily blends of high and low protein diets to precisely match estimated lysine and energy requirements. CON sows received the same blend and quantity of the high and low protein diets on each day of gestation.

^3^ Pregnancy.

^4^ 105 sows (average reproductive cycle 1.4 ± 0.48) reached the end of gestation in the first pregnancy, 73 sows (average reproductive cycle 2.4 ± 0.49) were successfully re-bred and reached the end of gestation in the second pregnancy, and 49 sows (average reproductive cycle 3.4 ± 0.50) were successfully re-bred and reached the end of gestation in the third pregnancy.

^5^ SID = standardized ileal digestible.

Upon entering the farrowing crate on approximately day 110 of gestation, sows received 2 kg of a standard lactation diet (Table 1) until farrowing on day 116 ± 1 of gestation. After farrowing, lactation feed allowance was increased in a stepwise manner until ad libitum feed intake was achieved around day 4. Lactation feed disappearance was recorded weekly after farrowing to calculate ADFI over the entire lactation period.

### Experimental Procedures

Individual sows were weighed weekly in each gestation cycle using a floor scale before the daily feeding cycle began. Sow backfat and loin depths were measured at the P2 position (6.5 cm from the midline over the last rib) on days 5 ± 3, 62 ± 3, and 110 ± 1 of gestation and 3 d after weaning by a trained technician using a portable ultrasound machine with a 140 mm linear probe (Agroscan L, ECM Noveko International Inc., Angoulême, France).

On days 5 ± 3 and 108 ± 1 of gestation, 10 mL of blood were collected from each sow via sub-orbital sinus puncture into serum tubes (BD Vacutainers, Mississauga, ON, Canada). Blood samples were centrifuged at 3000 × *g* at room temperature for 15 min. Serum was aliquoted into microcentrifuge tubes and stored at −20 °C until further analysis. Serum from a subset of 20 sows (*n* = 10 per treatment) from each of three subsequent gestation cycles were analyzed at the University of Guelph Animal Health Laboratories (Guelph, ON, Canada) using a Cobas 6000 c501 biochemistry analyzer (Roche Diagnosis, Laval, QC, Canada) to determine serum concentrations of glucose, urea, beta-hydroxybutyric acid (BHBA), and non-esterified fatty acids (NEFA).

Immediately after birth and before first suckle, up to two piglets per sow were sacrificed for determination of physical and chemical body composition. Body weight and individual organ weights (i.e., liver, stomach, small intestine, and large intestine; unemptied) were collected. The organs and carcass were combined per pig in a plastic bag and frozen at −20 °C until further analysis (Miller et al., 2020).

### Nutrient Analyses

Representative feed subsamples for the HP and LP diets were collected monthly (to include each batch of feed) and compiled into 3.5-mo intervals for analysis. Samples were ground and sent to SGS (SGS Canada Inc., Guelph, Ontario, Canada) and analyzed for dry matter (method 930.15), crude protein (method 990.03), and calcium and phosphorus (method 985.01; AOAC, 2005). Piglet carcasses (with organs) were removed from the freezer and sliced into 2.54- × 2.54-cm sections using a band saw. The sections were then passed through a meat grinder with a 1-cm screen, three times. After the third pass, a representative sub-sample of approximately 250 g was placed into an aluminum tin and stored at −20 °C; the remainder was discarded. Ground carcass samples were freeze-dried for 72 h and ground with a coffee grinder (Custom Grind Coffee Grinder, Hamilton Beach Brands Canada Inc., Belleville, ON, Canada) until a uniform particle size was achieved (Miller et al., 2020). Dry matter (method 930.15; AOAC, 2005) and ash (method 942.05; AOAC, 2005) contents were determined. Protein content (N × 6.25) was determined by combustion analysis (LECO-FP 828 analyzer, LECO Instruments Ltd., Mississauga, ON, Canada).

### Calculations and Statistical Analysis

Whole body mass balance calculations during gestation in each pregnancy cycle were estimated for N and P using equations from the NRC (2012) to determine maternal empty body weight, maternal body protein, weight of conceptus, protein contents of fetuses, placenta and associated fluids, uterus, and mammary gland, as well as P content for fetuses and placenta, and P retention in maternal body (Eq. 8-49, 8-51, 8-54, 8-56, 8-57, 8-59, 8-60, 8-67, 8-68, 8-69; NRC, 2012). Results were summed to calculate N (protein/6.25) and P contents, accordingly, and the difference between the end (days 108 ± 1) and beginning (days 5 ± 2) of gestation was used to determine N and P retention. Dietary N and P intake per gestation cycle was calculated based on individual sow (daily) feed recipes during the gestation period and analyzed crude protein and P contents (Table 1) and excesses were calculated as the difference between intake and retention. For calculation of feed costs, current ingredient prices were obtained from a commercial feed company (Wallenstein Feed Supply Ltd., Wallenstein, ON, Canada).

The experimental design was pre-planned to compare outcomes between treatments within pregnancies. Statistical analyses for gestation, farrowing, and lactation performance data were conducted using the GLIMMIX procedure of SAS (University Edition; SAS Inst. Inc., Cary, NC) with individual sow as the experimental unit. The model included the fixed effects of gestation feeding program treatment (treatment; TRMT), pregnancy number (PREG), and their interaction; initial reproductive cycle (INTPARITY) was used as a covariate and the repeated measures option was employed. The random effects of block, treatment nested within block, and sow nested within treatment were included. Piglet performance during the lactation period (after cross-fostering) was analyzed with the additional covariate of post-fostering litter size. Sow serum concentrations for BHBA, glucose, and NEFA were log transformed prior to analysis and back transformed for presentation of results. Pre-planned contrasts were constructed to compare treatments within pregnancy cycle. A *P*-value of *<*0.05 was considered significant, whereas values of 0.05 ≤ *P ≤* 0.10 were considered a trend.

## RESULTS

The analyzed and calculated nutrient contents were comparable for both the high and low protein gestation diets, as well as the commercial lactation diet (Table 1). The only exception was for analyzed crude protein content for the low protein gestation diet, which was 12% greater than the calculated value. There were differing levels for feed, energy, and Lys usage since sows that received the PF program had differing feed allowance and blend composition within and among pregnancies, whereas sows fed the CON program had the same daily feed allowances and blend composition, regardless of pregnancy number (Table 2).

The main effect of pregnancy is not mentioned in the results section since some variation in sows from different pregnancies was expected; the main effect *P-*values are presented in the tables for the reader’s information. Initial and final sow body weights and ADG between gestation days 5 and 37 were not influenced by gestation feeding program for any of the three pregnancies (Table 3). Between gestation days 38 and 72, ADG was influenced by the interaction of gestation feeding program and pregnancy (*P* < 0.05) where sows that received the PF program had greater ADG in pregnancies 2 and 3 than sows that received the CON program (contrasts; *P* < 0.05), whereas gestation feeding program did not affect ADG in pregnancy 1. Between gestation days 73 and 108, sows that received the PF program tended (*P* = 0.063) to have greater ADG than those that received the CON program; in pregnancy 1 only, sows fed the PF program had greater ADG than those fed the CON program (contrast; *P* < 0.05). Generally, backfat and loin depth values, along with changes in backfat and loin depths over gestation, were not influenced by gestation feeding program in any of the pregnancies. However, the loin depth gain between days 63 and 110 of gestation was greater for sows that received the PF versus the CON program (*P* < 0.05); in pregnancy 3, sows fed the PF program had greater loin depth gain than those fed the CON program (contrast; *P* = 0.072). The costs for the HP and LP diet usage per sow were less and greater, respectively, for sows that received the PF versus the CON program (*P* < 0.001 and *P* < 0.001). In pregnancies 1, 2, and 3, sows fed the PF program had lower feed cost associated with feeding the HP diet (contrasts; *P* = 0.077, *P* < 0.001, and *P* < 0.001, respectively) and greater feed cost associated with feeding the LP diet (contrasts; *P* < 0.05, *P* < 0.001, and *P* < 0.001, respectively). Cumulative feed cost (HP + LP) over the gestation period was greater for sows that received the PF vs. the CON program (*P* < 0.05). In pregnancies 1 and 2, cumulative feed cost tended to be (contrast; *P* = 0.060) and was (contrast; *P* < 0.001), respectively, greater for sows fed the PF vs. CON feeding program, but in pregnancy 3, cumulative gestation feed cost was not different between the feeding programs.

**Table 3.** Gestation performance for sows that received a precision (PF) or static (CON) feeding program during gestation over three consecutive pregnancies^1^

	Pregnancy 1		Pregnancy 2		Pregnancy 3			*P-*value 2		
Item	PF	CON	PF	CON	PF	CON	SEM^3^	TRMT	PREG	TRMT × PREG
No.^4^	50	55	36	37	25	24				
Body weight, kg										
Day 5	149.0	153.5	184.2	186.0	210.8	208.5	4.9	0.825	<0.001	0.369
Day 108	210.1	213.0	242.3	233.0	260.4	252.0	6.7	0.408	<0.001	0.205
ADG, g/d										
Days 5 to 37	182	287	13	−12	9	144	90	0.354	<0.001	0.412
Days 38 to 72	633	667	667*	522	541*	364	63	0.023	<0.001	0.019
Days 73 to 108	656*	513	762	672	738	668	94	0.063	0.028	0.793
Backfat depth, mm										
Initial	14.8	15.4	14.3	15.2	16.3	15.3	1.0	0.854	0.212	0.239
Final	16.2	16.5	17.7	17.9	19.6	18.7	1.1	0.871	<0.001	0.676
Gain: days 5 to 62	1.9	2.8	4.0	2.5	3.2	2.6	1.6	0.811	0.265	0.107
Gain: days 63 to 110	−0.5	−1.7	−0.8	0.3	0.0	0.7	1.5	0.908	0.068	0.093
Loin depth, mm										
Initial	69.5	68.4	68.9	70.1	70.2	69.4	1.5	0.744	0.473	0.175
Final	70.3	69.3	71.3	69.8	72.0	70.4	1.6	0.208	0.290	0.916
Gain: d 5 to 62	−1.1	0.5	2.9	0.8	2.0	4.8	1.7	0.387	0.031	0.220
Gain: d 63 to 110	1.9	0.5	0.0	−0.6	0.2†	−3.6	1.6	0.013	0.131	0.571
Gestation feed cost, $/sow^5^										
High protein diet	43.48†	45.15	33.30*	45.30	24.08*	45.40	0.71	<0.001	<0.001	<0.001
Low protein diet	28.54*	25.10	41.74*	24.94	46.79*	24.84	1.09	<0.001	<0.001	<0.001
Cumulative	72.04†	70.24	75.04*	70.19	71.06	70.16	0.80	0.013	0.001	0.001

^1^ Sows on the PF program received unique daily blends of high and low protein diets to precisely match estimated Lys and energy requirements. CON sows received the same blend and quantity of the high and low protein diets on each day of gestation. Upon entering farrowing crates (day 110 ± 1.4), sows received 2 kg per day of standard lactation diet until the day of farrowing (day 116±1.4). Thereafter, sows received increasing allotments of lactation diet until ad libitum intake was achieved on day 4 of lactation.

^2^ *P*-values for the main effects of gestation feeding program (TRMT), pregnancy (PREG), and the interactive effect of feeding program and pregnancy (TRMT × PREG).

^3^Maximum value for the standard error of the means.

^4^ 105 sows (average reproductive cycle 1.4 ± 0.48) reached the end of gestation in the first pregnancy, 73 sows (average reproductive cycle 2.4 ± 0.49) were re-bred and reached the end of gestation in the second pregnancy, and 49 sows (average reproductive cycle 3.4 ± 0.50) were re-bred and reached the end of gestation in the third pregnancy.

^5^ Calculated using commodity prices: corn: $220/ton; soybean meal: $445/ton; soy hulls: $250/ton.

* Values for PF sows are different from CON sows within pregnancy (*P* < 0.05).

† Values for PF sows tended to differ from CON sows within pregnancy (0.05 ≤ *P* ≤ 0.10).

The serum concentrations for BHBA and urea were not influenced by gestation feeding program in any pregnancy on either day 5 (initial) or day 108 (final) of gestation (Table 4). The serum concentrations for NEFA and glucose were not influenced by gestation feeding program on day 5 of gestation (initial) in any pregnancy. Serum NEFA concentration tended to be less for sows that received the PF vs. the CON program on day 108 of gestation (*P* = 0.071). Serum glucose tended to be influenced by the interactive effect of gestation feeding program and pregnancy (*P =* 0.059) where PF sows had greater serum glucose concentration on day 108 of gestation in pregnancy 2 (*P* < 0.05), whereas no differences in sercum glucose between gestation feeding programs were observed for pregnancies 1 or 3.

**Table 4.** Serum metabolites during gestation for sows that received a precision (PF) or static (CON) feeding program during gestation over three consecutive pregnancies^1^

	Pregnancy 1		Pregnancy 2		Pregnancy 3			*P-*value^2^		
Item	PF	CON	PF	CON	PF	CON	SEM^3^	TRMT	PREG	TRMT × PREG
No.^4^	10	10	10	10	10	10				
BHBA^5^, mmol/L										
Day 5	3.2	1.9	2.4	5.3	8.8	8.1	2.9	0.893	0.099	0.288
Day 108	1.9	33.0	7.4	21.9	4.1	40.0	19.4	0.247	0.615	0.980
NEFA^6^, mmol/L										
Day 5	0.2	0.1	0.1	0.1	0.1	0.0	0.04	0.250	0.060	0.698
Day 108	0.3	0.5	0.2	0.6	0.2	0.4	0.2	0.071	0.225	0.899
Glucose, mmol/L										
Day 5	3.9	3.9	4.1	3.8	4.9	4.6	0.3	0.310	0.002	0.802
Day 108	3.2	3.5	3.2*	2.7	3.4	3.2	0.2	0.349	0.026	0.059
Urea, mmol/L										
Day 5	3.7	3.5	4.0	3.9	5.1	5.1	0.4	0.824	<0.001	0.882
Day 108	4.0	3.4	3.5	3.2	2.9	3.0	0.3	0.450	0.002	0.137

^1^ Sows on the PF program received unique daily blends of high and low protein diets to precisely match estimated Lys and energy requirements. CON sows received the same blend and quantity of the high and low protein diets on each day of gestation. Upon entering farrowing crates (day 110±1.4), sows received 2 kg per day of standard lactation diet until the day of farrowing (day 116±1.4). Thereafter, sows received increasing allotments of lactation diet until ad libitum intake was achieved on day 4 of lactation.

^2^ *P*-values for the main effects of gestation feeding program (TRMT), pregnancy (PREG), and the interactive effect of feeding program and pregnancy (TRMT × PREG).

^3^Maximum value for the standard error of the means.

^4^ Blood samples were collected from the same 20 sows in pregnancies 1, 2, and 3 on day 5 ± 2.5 after breeding (initial) and day 108 ± 1.4 of gestation (final).

^5^ Beta-Hydroxybutyric acid.

^6^ Non-esterified fatty acids.

* Values for PF sows are different from CON sows within pregnancy (*P* < 0.05).

†Values for PF sows tended to differ from CON sows within pregnancy (0.05 ≤ *P* ≤ 0.10).

The number of piglets born alive, piglet birth weight, the variation in piglet birth weights before and after cross-fostering, litter birth weight, and the number of stillbirths were not influenced by gestation feeding program in any pregnancy (Table 5). The number of mummified piglets tended to be greater for sows that received the PF program in pregnancy 3 only (contrast; *P* = 0.092). Piglet BW, liver, and large intestine mass, and relative gastrointestinal tract weight (% of BW) at birth were not influenced by gestation feeding program in any pregnancy (Table 6). Piglet stomach mass was influenced by the interaction between gestation feeding program and pregnancy (*P* < 0.01), such that piglets from sows that received the PF program had greater stomach mass in pregnancy 3 (contrast; *P* < 0.05) but stomach masses were not different between feeding programs in pregnancies 1 or 2. Piglets from sows that received the PF program tended to have greater small intestine mass than piglets from sows that received the CON program in pregnancy 2 only (contrast; *P* = 0.057). The protein content in piglet carcasses was less for sows that received the PF vs. the CON program (*P* < 0.05), which was especially evident in pregnancy 3 (contrast; *P* < 0.05). The piglet ash and fat contents were not influenced by gestation feeding program in any pregnancy but the water content was greater for piglets from sows that received the PF vs. the CON program (*P* < 0.05).

**Table 5.** Litter characteristics at birth for sows that received a precision (PF) or static (CON) feeding program during gestation over three consecutive pregnancies^1^

	Pregnancy 1		Pregnancy 2		Pregnancy 3			*P-*value 2		
Item	PF	CON	PF	CON	PF	CON	SEM^3^	TRMT	PREG	TRMT × PREG
No.^4^	50	55	36	37	25	24				
Born alive, no.	11.0	12.0	11.3	11.0	12.3	11.5	0.7	0.995	0.376	0.181
Piglet birth weight, kg^5^	1.4	1.4	1.5	1.5	1.4	1.5	0.1	0.594	0.001	0.439
CV piglet BW at birth, %^5^	19.3	19.9	19.5	21.0	21.6	21.2	1.5	0.652	0.329	0.731
CV piglet BW post-foster, %	18.0	18.4	19.6	21.3	21.8	22.6	1.5	0.336	0.002	0.791
Litter birth weight, kg^5^	14.7	16.3	16.6	16.9	16.6	17.7	1.0	0.300	0.023	0.541
Stillborn, no.	0.8	0.8	0.8	1.1	1.1	0.8	0.3	0.232	0.984	0.354
Mummified, no.	0.2	0.2	0.2	0.2	0.4†	0.2	0.1	0.212	0.461	0.356

^1^ Sows on the PF program received unique daily blends of high and low protein diets to precisely match estimated Lys and energy requirements. CON sows received the same blend and quantity of the high and low protein diets on each day of gestation. Upon entering farrowing crates (day 110 ± 1.4), sows received 2 kg per day of standard lactation diet until the day of farrowing (day 116 ± 1.4). Thereafter, sows received increasing allotments of lactation diet until ad libitum intake was achieved on day 4 of lactation.

^2^ *P*-values for the main effects of gestation feeding program (TRMT), pregnancy (PREG), and the interactive effect of feeding program and pregnancy (TRMT × PREG).

^3^Maximum value for the standard error of the means.

^4^ 105 sows (average reproductive cycle 1.4 ± 0.48) reached the end of gestation in the first pregnancy, 73 sows (average reproductive cycle 2.4 ± 0.49) were re-bred and reached the end of gestation in the second pregnancy, and 49 sows (average reproductive cycle 3.4 ± 0.50) were re-bred and reached the end of gestation in the third pregnancy.

^5^ Did not include BW of stillborn piglets.

^*^ Values for PF sows are different from CON sows within pregnancy (*P* < 0.05).

† Values for PF sows tended to differ from CON sows within pregnancy (0.05 ≤ *P* ≤ 0.10).

**Table 6.** Physical and chemical body composition of piglets at birth for sows that received a precision (PF) or static (CON) feeding program during gestation over three consecutive pregnancies^1^

	Pregnancy 1		Pregnancy 2		Pregnancy 3			*P-*value 2		
Item	PF	CON	PF	CON	PF	CON	SEM^3^	TRMT	PREG	TRMT × PREG
No.^4^	17	17	16	10	7	4				
Birth weight, kg	1.3	1.4	1.4	1.4	1.4	1.4	0.1	0.445	0.224	0.575
Organ weight, g^5^										
Liver	51.5	48.5	45.5	48.0	46.5	42.7	5.5	0.645	0.259	0.525
Stomach	11.1	12.4	10.0	10.5	15.5*	7.8	2.2	0.026	0.251	0.002
Small intestine	56.9	59.9	63.9†	54.2	59.5	57.9	6.1	0.428	0.980	0.144
Large intestine	13.9	14.8	14.3	15.7	15.8	18.5	0.1	0.115	0.143	0.796
GIT, % BW^6^	61.6	64.8	62.2	57.5	66.2	58.6	5.9	0.482	0.423	0.258
Chemical body composition, % BW										
Protein	11.0	11.0	10.6	10.9	8.9*	10.4	0.5	0.049	0.009	0.114
Ash	4.0	4.2	3.9	3.9	3.2	3.6	0.3	0.363	<0.001	0.762
Fat	0.7	0.7	0.6	0.7	0.6	0.8	0.1	0.205	0.247	0.327
Water	85.8	83.5	84.8	84.0	87.9	85.5	1.3	0.028	0.102	0.513

^1^ Sows on the PF program received unique daily blends of high and low protein diets to precisely match estimated Lys and energy requirements. CON sows received the same blend and quantity of the high and low protein diets on each day of gestation. Upon entering farrowing crates (day 110 ± 1.4), sows received 2 kg per day of standard lactation diet until the day of farrowing (day 116 ± 1.4). Thereafter, sows received increasing allotments of lactation diet until ad libitum intake was achieved on day 4 of lactation.

^2^ *P*-values for the main effects of gestation feeding program (TRMT), pregnancy (PREG), and the interactive effect of feeding program and pregnancy (TRMT × PREG).

^3^Maximum value for the standard error of the means.

^4^ Piglets were sampled from 10, 9, and 6 PF sows and 9, 5, and 2 CON sows in pregnancies 1, 2, and 3, respectively.

^5^ Liver weight excluded the gallbladder; gastrointestinal tract segments were weighed including gut contents.

^6^ Gastrointestinal tract weight (sum of stomach, small intestine, and large intestine) as a percentage of live piglet body weight.

^*^ Values for PF sows are different from CON sows within pregnancy (*P* < 0.05).

^†^ Values for PF sows tended to differ from CON sows within pregnancy (0.05 ≤ *P* ≤ 0.10).

There was an interactive effect of sow feeding program and pregnancy on the number of piglets per litter after cross-fostering (*P* < 0.01). In pregnancy 1, the litter size for sows that received the PF program tended to be smaller than the litter size for CON-fed sows (contrast; *P* = 0.059), whereas litter size was greater in pregnancies 2 and 3 for sows that received the PF program (contrasts; *P* < 0.05; Table 7). The number of piglets weaned, piglet weaning weights, and piglet ADG during the lactation period were not influenced by gestation feeding program in any pregnancy. There was an interactive effect of sow feeding program and pregnancy on the coefficient of variation for piglet BW at weaning (*P* < 0.001), such that the variation was greater for piglets from sows that received the PF program in pregnancy 1 (contrast; *P* < 0.05), was less in pregnancy 2 (contrast; *P* < 0.01), and not different in pregnancy 3 vs. piglets from sows that received the CON program. There was an interactive effect of feeding program and pregnancy on sow BW change over lactation (*P* < 0.05). In pregnancy 2, sows that received the PF program tended to lose less BW than sows that received the CON feeding program (contrast; *P* = 0.091), but no differences in sow BW loss between feeding programs were observed in pregnancies 1 and 3. Changes in backfat and loin depths over lactation and re-breeding interval were not influenced by gestation feeding program in any pregnancy. Average daily feed intake and cumulative feed cost during lactation were less for sows that received the PF versus CON program (*P* < 0.01), which was especially apparent in pregnancy 2 (contrast; *P* < 0.05).

**Table 7.** Lactation performance for sows that received a precision (PF) or static (CON) feeding program during gestation over three consecutive pregnancies^1^

	Pregnancy 1		Pregnancy 2		Pregnancy 3			*P-*value 2		
Item	PF	CON	PF	CON	PF	CON	SEM3	TRMT	PREG	TRMT × PREG
No.^4^	50	55	36	37	25	24				
Piglets										
Post-fostering, no.	10.0†	10.6	11.2*	10.4	11.4*	10.3	0.4	0.078	0.078	0.004
Weaned, no.	9.7	9.9	9.8	9.9	9.8	10.0	0.3	0.396	0.920	0.978
Wean weight, kg	6.4	6.5	6.8	6.9	6.7	6.6	0.2	0.640	0.008	0.664
CV piglet BW at weaning, %	17.7*	15.6	15.2*	19.3	17.3	18.6	1.1	0.179	0.297	<0.001
ADG, g/d	243	235	255	272	248	249	10	0.630	0.003	0.238
Sow										
BW change, kg	−8.6	−8.4	−10.2†	−16.9	−14.9	−8.5	3.5	0.997	0.065	0.032
Backfat change, mm	−2.3	−1.2	−2.1	−3.6	−1.9	−3.4	1.0	0.347	0.364	0.115
Loin depth change, mm	−3.1	−0.7	−6.7	−4.7	−2.1	−5.1	2.6	0.743	0.098	0.353
Average daily feed intake, kg	5.4	5.7	5.9*	6.7	5.8	6.1	0.2	0.008	<0.001	0.163
Feed cost, $/sow^5^	37.60	39.72	38.51*	43.14	38.90	41.04	1.74	0.031	0.139	0.469
Re-breeding interval, d	4.9	4.9	4.5	4.3	4.0	4.3	0.3	0.765	<0.001	0.494

^1^ Sows on the PF program received unique daily blends of high and low protein diets to precisely match estimated Lys and energy requirements. CON sows received the same blend and quantity of the high and low protein diets on each day of gestation. Upon entering farrowing crates (day 110 ± 1.4), sows received 2 kg per day of standard lactation diet until the day of farrowing (day 116 ± 1.4). Thereafter, sows received increasing allotments of lactation diet until ad libitum intake was achieved on day 4 of lactation.

^2^ *P*-values for the main effects of gestation feeding program (TRMT), pregnancy (PREG), and the interactive effect of feeding program and pregnancy (TRMT × PREG).

^3^Maximum value for the standard error of the means.

^4^ 105 sows (average reproductive cycle 1.4 ± 0.48) reached the end of gestation in the first pregnancy, 73 sows (average reproductive cycle 2.4 ± 0.49) were re-bred and reached the end of gestation in the second pregnancy, and 49 sows (average reproductive cycle 3.4 ± 0.50) were re-bred and reached the end of gestation in the third pregnancy.

^5^ Calculated using commodity prices: corn: $220/ton; barley: $285/ton; wheat: $285/ton; soybean meal: $445/ton; soy hulls: $250/ton.

* Values for PF sows are different from CON sows within pregnancy (*P* < 0.05).

† Values for PF sows tended to differ from CON sows within pregnancy (0.05 ≤ *P* ≤ 0.10).

Estimated N and P retentions were not influenced by gestation feeding program in any pregnancy (Table 8). There was an interactive effect of sow feeding program and pregnancy on excess N (*P =* 0.050), such that sows that received the PF program had lower excretion of excess N in pregnancies 2 and 3 vs. sows that received the CON program (contrasts; *P <* 0.05 and *P <* 0.01, respectively), whereas there was no difference in excess N in pregnancy 1. Overall, sows that received the PF program had lower amounts of excess N compared to sows that received the CON program (*P* < 0.01). Sows that received the PF program had greater excretion of excess P compared to sows that received the CON program (*P* < 0.01), particularly in pregnancies 2 and 3 (contrasts; *P <* 0.05 and *P <* 0.01, respectively).

**Table 8.** Estimated nitrogen and phosphorus retention for sows that received a precision (PF) or static (CON) feeding program during gestation over three consecutive pregnancies^1^

	Pregnancy 1		Pregnancy 2		Pregnancy 3			*P-*value 2		
Item	PF	CON	PF	CON	PF	CON	SEM^3^	TRMT	PREG	TRMT × PREG
No.	46	52	33	35	23	22				
Nitrogen										
Retained, % of intake	27.4	28.3	27.4	24.3	25.6	23.6	2.3	0.434	0.240	0.479
Excess, g/sow	1711	1680	1622*	1779	1517*	1791	54	0.001	0.592	0.050
Phosphorus										
Retained, % of intake	16.0	16.3	14.3	12.7	10.3	12.3	1.9	0.840	0.013	0.568
Excess, g/sow	1433	1373	1535*	1430	1599*	1437	35	<0.001	<0.001	0.241

^1^ Sows on the PF program received unique daily blends of high and low protein diets to precisely match estimated Lys and energy requirements. CON sows received the same blend and quantity of the high and low protein diets on each day of gestation. Upon entering farrowing crates (day 110 ± 1.4), sows received 2 kg per day of standard lactation diet until the day of farrowing (day 116 ± 1.4). Thereafter, sows received increasing allotments of lactation diet until ad libitum intake was achieved on day 4 of lactation.

^2^ *P*-values for the main effects of gestation feeding program (TRMT), pregnancy (PREG), and the interactive effect of feeding program and pregnancy (TRMT × PREG).

^3^Maximum value for the standard error of the means.

* Values for PF sows are different from CON sows within pregnancy (*P* < 0.05).

## DISCUSSION

The purpose of the current study was to examine the long-term effects of closely meeting the dynamic estimated Lys and energy requirements for individual sows during gestation across three consecutive pregnancies on sow reproductive and lactation performance. Using the PF program, sows gained the same overall amount of BW during gestation, but with greater BW gain occurring after day 38 of gestation versus sows that received a static quantity and composition of gestation diet (CON). This, combined with positive and greater loin depth gains after day 63 of gestation and 7 and 2× (average over three pregnancies) lower day 108 fasted serum BHBA and NEFA concentrations, respectively, indicates that the dynamic and increasing SID Lys:NE ratio provided throughout gestation for the PF program, supplied adequate amounts of both Lys and energy to support maternal protein deposition and minimize lipid mobilization. However, since there were no differences in piglet birth weights, piglet ADG (indicator of sow milk production), re-breeding intervals, and sow retention, it appeared there were no negative long-term implications for CON-fed sows. In modeled scenarios, it has been demonstrated that a static SID Lys supply of 11 g/d throughout gestation resulted in maternal protein and lipid mobilization in late gestation, while providing 13.5 g/d SID Lys was above estimated requirements for both gilts and sows (Thomas et al., 2021). Furthermore, supplying additional energy when amino acids were not limiting between days 90 and 114 of gestation increased maternal protein retention for sows in the first, second, and third reproductive cycles (Miller et al., 2016; Miller et al., 2017). In the current study, sows that received the PF program consumed relatively less SID Lys per day (except after day 73 in the first and second pregnancies) but more net energy per day after day 37 of gestation (and especially after day 73). Thus, based on the Lys and NE intakes, the SID Lys:NE ratio was relatively less for the PF program in all pregnancies and throughout gestation, except after day 73 in the first pregnancy. Therefore, it is likely that the CON feeding program was limiting in energy for maternal protein and lipid deposition, particularly in late gestation.

Sows that received the PF program during gestation consumed less lactation diet in the subsequent lactation period vs. those that received the CON program. Since overall sow BW change and piglet ADG during lactation were not different between sows from the two gestation feeding programs, sows that received the PF program during gestation were presumably more efficient at utilizing dietary nutrients and energy for milk production. This occurred despite PF sows unintentionally tending to have larger litter sizes than CON sows after cross-fostering. During lactation, when feed intake is no longer restricted, sows attempt to compensate for nutrient and energy deficiencies experienced in gestation by increasing feed intake (Revell et al., 1998). Thus, sows that received the CON feeding program during gestation may have attempted to increase energy and nutrient intakes during lactation to maintain milk production and minimize further mobilization of maternal protein and energy stores.

In the current study, there were minimal differences in litter characteristics and piglet physical and chemical composition at birth from sows that received the PF and CON gestation feeding programs. Piglets from sows that received the PF program in gestation, however, had greater variation in BW within litters at weaning in pregnancy cycle 1, less variation in pregnancy cycle 2, and no difference in variation in pregnancy cycle 3, despite no differences in BW variation within litters at birth or after cross-fostering. Variation in BW at weaning can be linked to poor lactation output (Lee et al., 2014); however, there were no differences in the overall piglet ADG during the lactation period or average BW at weaning, indicating that milk production per piglet was comparable between sows that received the PF and CON gestation feeding programs. In addition, piglets were allowed access to creep feed to mimic a commercial environment, and the disappearance of creep feed was not monitored among litters, which could have influenced BW distribution within litters for piglets that did and did not consume creep feed. In terms of physical body composition, only the stomach mass for newborn piglets was influenced by maternal gestation feeding program, but this was likely due to the low number of replicates per treatment, particularly in the third pregnancy. Finally, in terms of piglet chemical body composition at birth, all components were within the range indicated by others (e.g., Miller et al., 2020) and were minimally influenced by maternal gestation feeding program, which provides further evidence that the sow was able to buffer dietary supply of nutrients and energy to ensure the developing fetuses were not influenced under the conditions of the current study.

Overall, gestation feeding program had no obvious effect on sow retention throughout the study, although the number of sows enrolled was relatively small. Nearly equal numbers of sows were removed from the study for: lameness (1 PF; 2 CON), reproductive failure (i.e., failure to enter estrus after weaning, failure to conceive when inseminated, miscarriage after pregnancy confirmation, poor litter size, or failure to produce milk; 14 PF; 20 CON), illness (7 PF; 4 CON), savaging piglets (1 PF; 1 CON), death with unknown cause (2 PF; 1 CON), and incompatibility with the ESF system (0 PF; 2 CON). It is interesting to note that 30% more CON sows were removed from the study due to reproductive failure compared to PF sows, with the most common reproductive issues being failure to enter estrus after weaning, failure to conceive after insemination, and miscarriage. The reproductive issue of failing to return to estrus is typically due to low BW at the time of breeding, which is caused by BW loss during lactation and poor adipose and protein reserves at weaning (Clowes et al., 2003b; Koketsu et al., 2017). In the current study, the amount of BW lost during lactation and losses of backfat and loin depth were not different between sows that received the PF and CON gestation feeding programs. Moreover, no diseases that are known to cause disruptions in reproduction were diagnosed in the sow herd. Therefore, future studies should examine the effects of precision feeding during gestation on sow retention and possible implications for reproductive success.

The cumulative feed cost during gestation was greater for sows that received the PF vs. CON feeding program, which was largely driven by greater feed allowance (i.e., sum of HP and LP diets per sow in each pregnancy cycle) to meet estimated daily energy requirements. Considering the reduction in feed intake (and cost) during lactation for sows that received the PF program, the relative difference in feed cost per entire reproductive cycle was small (i.e. ~ $0.25 per sow in pregnancies 1 and 2). Conversely, in pregnancy 3, the sum of gestation and lactation feed costs per sow were $1.24 less for sows on the PF gestation feeding program. As sows mature, the estimated daily energy requirements increase slightly to account for greater maintenance needs, but AA requirements decrease as maternal protein retention approaches zero (once losses from the previous lactation are recuperated; NRC, 2012). Therefore, and depending on commodity prices, the feed cost for the PF program could become relatively less in higher parities, as was observed in the current study. Commercial gestating feeding programs are typically designed to meet the (average) estimated Lys requirements for sows in the first reproductive cycle and the same diet is fed to sows for all parities, which results in over-feeding Lys (protein) to older sows (Thomas et al., 2021). Indeed, the estimated amount of excess N was not different between the PF and CON feeding programs in pregnancy 1, but in pregnancies 2 and 3, excess N was reduced for the sows that received the PF program. This supports the work of others which demonstrated that phase- or precision-feeding gestating sows can reduce both nitrogen intake and excretion, without negatively affecting sow performance (Clowes et al., 2003a; Gaillard et al., 2020). In addition, in the current study, excess P was greater for sows that received the PF vs. CON program, despite similar retention efficiencies that were comparable to those reported by others (e.g., van der Peet-Schwering et al., 1999; Buis, 2016). The LP and HP diets contained the same P contents and since the PF program provided a greater feed allowance to sows, P intake and excretion were also greater. If P was provided more precisely (i.e., scaled with Lys in the HP and LP diets or by including a third diet to the blend), then there is also further opportunity to reduce feed costs as well as P excretion to the environment (Gaillard et al., 2020). Finally, the cost of the PF program will also be largely influenced by commodity prices and ingredient selection. For example, when the price of protein sources increases, the PF program may become more favorable. Ultimately, evaluating the return-on-investment of precision feeding gestating sows will depend on individual farm factors, but should include the benefits of minimizing over-feeding N, reducing excess N excretion from the farm (where incentives are available; e.g., van der Peet-Schwering et al., 1999), and potentially, reducing lactation feed costs.

## CONCLUSION

Using feeding programs that precisely match estimated daily energy and Lys requirements for gestating sows provides the opportunity to reduce nitrogen losses to the environment and reduce lactation feed usage, without negatively affecting sow reproductive and lactation performance. Based on the conditions of the current study with the genetics used across three consecutive gestation cycles, there appeared to be no ill effects for sows that received a static amount of energy and Lys throughout gestation and across parities. Although there were no notable differences in piglet characteristics from the time of birth to weaning, it is not known if precisely meeting estimated energy and Lys requirements throughout gestation will influence offspring post-weaning growth performance. Depending on commodity prices and individual farm factors, implementing a precision feeding program in gestation could be a means to reduce gestation and lactation feed costs. Future studies should examine the impact of precision feeding gestating sows in commercial scenarios using modern, hyper-prolific sow genetics.
